# Endoscopic endonasal transsphenoidal approach improves endocrine function and surgical outcome in primary craniopharyngioma resection: a systematic review and meta-analysis

**DOI:** 10.1186/s12957-024-03411-8

**Published:** 2024-05-24

**Authors:** Shuang Li, Youfan Ye, Chuansheng Nie, Xing Huang, Kaixuan Yan, Fangcheng Zhang, Xiaobing Jiang, Haijun Wang

**Affiliations:** 1 Department of Neurosurgery, Union Hospital, Tongji Medical College, Huazhong University of Science and Technology, 1277# Jiefang Avenue, Wuhan, 430022 Hubei China; 2 Department of Ophthalmology, Union Hospital, Tongji Medical College, Huazhong University of Science and Technology, 1277# Jiefang Avenue, Wuhan, 430022 Hubei China

**Keywords:** Meta-analysis, Microscopic transcranial approach, Endoscopic endonasal transsphenoidal approach, Craniopharyngiomas, Neurosurgery

## Abstract

**Background:**

Craniopharyngiomas (CPs) are generally derived from the craniopharyngeal duct epithelium, accounting for 38% and 24.5% of mortality in pediatric and adult patients, respectively. At present, the widespread application of the endoscopic endonasal transsphenoidal approach (EEA) has led to controversy between the traditional microscopic transcranial approach (TCA) and EEA in relation to the surgical management of CPs.

**Object and method:**

We performed a systematic review and meta-analysis comparing the complications, surgical outcomes, and endocrine functions of patients with CPs to provide evidence-based decision-making in their surgical management.

**Result:**

Overall, 11 observational studies with 12,212 participants were included in the meta-analysis, in which five of them only included an adult population, three of them only included a child population, and the other three studies included a mixed population (adult and child). In pediatric patients, the EEA achieved a higher gross total resection (GTR) rate (odds ratio (OR) = 5.25, 95%CI: 1.21–22.74), lower recurrence rate (OR = 0.54, 95%CI: 0.31–0.94, *p* = 0.030), and less hypopituitarism (OR = 0.34, 95%CI: 0.12–0.97, *p* = 0.043). In adult patients, EEA significantly improved mortality (OR = 0.09, 95%CI: 0.06–0.15, *p* < 0.001) and visual outcomes (visual improvement: OR = 3.42, 95%CI: 1.24–9.40, *p* = 0.017; visual deficit: OR = 0.30, 95%CI: 0.26–0.35) with decreases in postoperative stroke (OR = 0.58, 95%CI: 0.51–0.66, *p* < 0.001), hydrocephalus, and infections (OR = 0.32, 95%CI: 0.24–0.42, *p* < 0.001).

**Conclusion:**

Compared with the traditional TCA in primary CP resection, the development and wide application of EEA optimistically decreased the recurrence rate of CP, alleviated hypopituitarism with improvement in the GTR rate of pediatric patients, and significantly improved the visual outcomes, hydrocephalus, postoperative stroke, survival, and infection rates of the patients. Therefore, EEA is an optimal approach for primary CP resection.

**Supplementary Information:**

The online version contains supplementary material available at 10.1186/s12957-024-03411-8.

## Introduction

Craniopharyngiomas (CPs) constitute 1.2–4.6% of all intracranial tumors, accounting for 0.5–2.5 new cases per 1 million population per year globally [1]. As a slow-growing benign tumor of the central nervous system, CPs generally originate from the hypothalamic-pituitary axis and develop from Rathke’s pouch [2, 3]. Approximately 50% of CPs can be found along the hypothalamic-pituitary axis and tuber cinereum at the level of the bottom of the third ventricle and develop primarily toward the third ventricle, which brings CPs to the possibility of being surrounded by several vital structures, such as hypothalamus, optic chiasm, and pituitary gland [1, 2, 4]. Therefore, the surgical management of CPs still remains a great challenge in clinical practice [2, 5].

Although surgical resection is considered the primary treatment for CPs, the most optimal surgical treatment approach has not been fully established. Generally, CPs are commonly excised via traditional microscopic TCA. Recently, the application of EEA in tumors, which are resected through a transsphenoidal procedure, has become more important. Over the past decades, with the advancement of neurosurgery instruments, the EEA has gradually surpassed traditional microscopic TCA for the surgical management of CPs [6, 7]. This prior effect may be attributed to the fact that the EEA provides significant improvement in the GTR rate and postoperative hypopituitarism, providing a more direct visualization of these tumors [8, 9]. Although several evidences support that the EEA increased the cerebrospinal fluid (CSF) leakage rate, less occurrence was observed in the visual deterioration, diabetes insipidus, tumor recurrence, and recurrence-free survival rate. However, in consideration of the complexity of this condition, some literatures identified distinct conclusions, including a similar GTR rate and equal recurrence rate between the EEA and TCA. Therefore, further research is needed to confirm the safety and acceptability of the EEA in this disputable field [10].

At present, only a few meta-analyses comparing the EEA and TCA were published, which were limited to mixing adults and pediatric patients or synthesizing effect size of single proportions rather than dichotomous outcomes [8, 11–16]. It is well-accepted that there is a limitation of the methodology of statistical analysis in single-proportion meta-analysis, because a single-proportion meta-analysis commonly ignores the heterogeneity of the included study when pooling proportions and drawing conclusions, which might lead to a lack of interpretability and misleading conclusions [17–19]. Moreover, it is well-known that the clinical outcomes of the GTR rate, survival rate, pituitary hormone deficits, and incidence of hypothalamic-pituitary dysfunction disorders might be different between primary and repeat craniopharyngioma resection, whereas in these previous studies, the definition of the type of craniopharyngioma was not established (primary, recurrence or mixed) [20–22].

To summarize the above arguments, a large-scale comparative meta-analysis including current available studies might present better evidence in the field. In this study, we aim to provide the latest evidence by performing a systematic review and meta-analysis to quantificationally and comprehensively evaluate the safety and effectiveness of EEA and TCA in primary CP resection according to the stratification of different age groups (i.e., child, adult, and mixed populations).

## Method

### Data sources and search strategy

This study was conducted by following the Preferred Reporting Items for Systematic Reviews and Meta-Analysis guidelines (PRISMA) [23], Assessing the methodological quality of systematic reviews Guidelines (AMSTAR) [24] and the Cochrane Collaboration’s systematic review framework. We performed a comprehensive search on PubMed, Ovid, and Cochrane Library databases to include potentially eligible studies. The search strategy was determined via the following items: (1) endoscopic endonasal; (2) (endoscopic endonasal) OR (endoscopic transsphenoidal) OR (transsphenoidal OR transcranial); (3) 1 OR 2; (4) craniopharyngioma; (5) (craniopharyngioma OR (Rathkes Pouch Tumor) OR (Rathke Pouch Tumor) OR (Rathkes Cleft Neoplasm) OR (Rathke Cleft Neoplasm) OR (Papillary Craniopharyngioma) OR (Child Craniopharyngioma) OR (Adamantinomatous Craniopharyngioma) OR (Adamantinomatous Craniopharyngiomas) OR (hypophyseal duct tumor) OR (adamantinoma) OR (adamantinomas) OR (Craniopharyngeal duct tumour) OR (Adamantinomatous tumour)); (6) 4 OR 5; (7) 3 AND 6.

### Study identification and exclusion

The study comprising a detailed description of surgical outcomes for comparation between EEA and TCA in CPs patients was independently identified and reviewed by two experienced investigators. The third researcher would involve in the assessment, if the opposite judgment appears.

Inclusion criteria were as follows: (1) randomized controlled trials (RCTs) or observational studies investigating surgical outcomes of EEA as compared with TCA in CP resection; (2) patients were all diagnosed with primary CPs, not recurrently CPs; (3) postoperative outcomes were reported at least GTR and CSF leakage rate.

Exclusion criteria were as follows: (1) studies merely contained a single series; (2) populations included recurrent CPs; (3) surgical approaches were not EEA and TCA; (4) patients were treated with radiotherapy.

### Data extraction

The following information was extracted: first author, journal name, year of publication, baseline demographic (mean age, gender proportion, and the number of participants in each group), and postoperative clinical characteristics: (1) surgical outcomes: GTR, recurrence, visual improvement and visual deficit; (2) endocrine functions: diabetes insipidus and hypopituitarism; (3) complications: CSF leakage, hydrocephalus, infection, stroke (ischemic and hemorrhagic), all-cause mortality, and thrombosis (pulmonary embolism or deep venous thrombosis); (4) length of stay (LOS) and follow-up period. No unpublished data were received by the authors from the included studies.

Two investigators independently collected these concerning medical data in eligible studies.

### Quality assessment in individual studies

The methodological quality of included studies was independently evaluated by the consensus of two experienced investigators based on the Newcastle-Ottawa Scale (NOS) comprising 3 assessment items: selection, comparability, and outcomes. Studies that achieved six or more stars on the modified NOS were considered high quality.

### Statistical analysis

To accurately analyze the statistical effect of various end point events, we calculated pooled odds ratio (OR) with a 95% confidence interval (CI) for dichotomous outcomes and the significance level was set to *P* < 0.05. The I [2] statistic (the significance level was set to *P* < 0.1) was used to assess the heterogeneity and determine the applicable effect model of each analysis. If I^2^ < 40%, the fixed-effect model was used, otherwise, the random-effect model was performed for the heterogeneity considered non-negligible. Stratified analyses were conducted according to different classification of age at admission (child, adult and mixed population). Egger’s test was performed to detect potential publication bias. All statistical analyses were conducted by Stata software 12.0.

## Results

### Characteristics of eligible studies

**Fig. 1:**
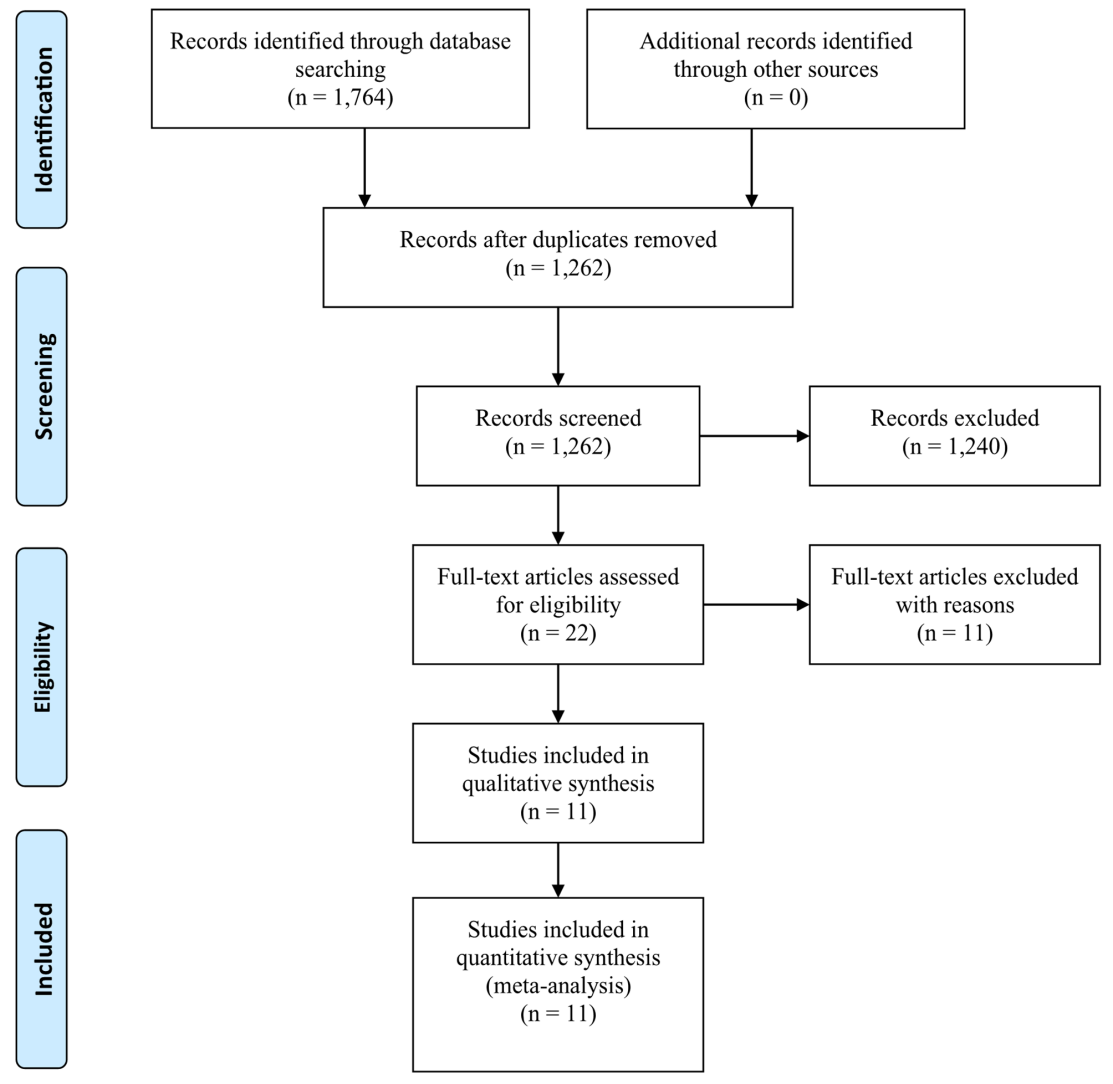
Flow diagram for the selection of included articles

A total of 1,764 potential publications were identified from PubMed, Ovid, and Cochrane Library databases until January 1, 2023, in which 502 publications were determined as duplicates. Two investigators independently reviewed the remaining 1,262 publications by reading titles, abstracts, and full texts. Eventually, 11 observational studies with 12,212 participants were considered qualified for our meta-analysis, 5 of them only included adult population, 3 of them only included child population, and the other 3 studies included mixed population (adult *plus* child) [16, 25–34]. The flowchart of literature search was shown in Fig. 1.

The majority of included studies were conducted in U.S.A or China, except 1 in India and 1 in Italy. In all 12,212 patients, EEA and TCA were respectively performed in 6,910 (56.7%) and 5,268 (43.3%) patients. 55% of included patients were female. The time span of all included studies was between 2016 and 2021. Table 1 shows the characteristic of included studies.

It is accepted that the anatomical relationship, the consistency and the volume of the tumor may influence the surgeon’s choice of surgical approach, we described the radiological characteristic of the included studies (Table 2). Two among the 11 included studies fail to report these data [18, 31]. In the studies which reported these original data, there were no significantly statistical differences in the anatomical location, consistency, and volume of the tumor between the EEA and TCA groups. Table 3 and 4 showed the conclusive results of meta-analysis and advantage of two surgical approach, respectively.

**Table 1:** Characteristics of included studies

Studies	Years	Countries	Populations	Number of		Presenting symptoms (EEA/TCA)			Mean of length of hospital stay (days)
				EEA/TCA	Male/Female	Headache	Visual deficits	Endocrinopathy	
**Gallotti**	2021	Italy	Adult	20/39	33/26	NA	14/27	NA	NA
**Govindarajan**	2021	USA	Adult	6511/4655	5466/5700	NA	NA	NA	3.7/7.0*
**Li**	2018	China	Adult	17/26	23/20	4/11	15/20	5/11	NA
**Wannemuehler**	2016	USA	Adult	9/12	8/13	6/5	8/10	4/3	10.1/14.4
**Moussazadeh**	2016	USA	Adult	21/5	19/7	4/1	15/4	7/1	9.3/15
**Konar**	2021	India	Child	14/28	24/18	11/24	7/23	1/2	4.2/7.4*
**Madsen**	2019	USA	Child	28/15	16/27	13/9	4/3	6/0	13.0/15.5
**Lin**	2017	USA	Child	100/100	90/108	NA	NA	NA	6.6/12.3
**Nie**	2022	China	Mixed	88/185	128/145	71/131	47/87	33/75	NA
**Fan**	2021	China	Mixed	125/190	184/131	67/100	84/124	44/63	NA
**Ozgural**	2018	Turkey	Mixed	11/13	9/15	NA	9/10	11/8	6/8*

**Notes**: EEA: endoscopic endonasal transsphenoidal approach; TCA: microscopic transcranial approach; NA: not available; *: median

**Table 2:** Characteristics of radiological data of the two surgical groups

Author	Years	Populations	*P* value of tumor topography	*P* value of tumor volume	*P* value of tumor consistency	*P* value of tumor calcification
**Gallotti**	2021	Adult	0.025	0.148	0.267	NA
**Govindarajan**	2021	Adult	NA	NA	NA	NA
**Li**	2018	Adult	1	0.146	0.075	1
**Wannemuehler**	2016	Adult	NA	0.16	1	1
**Moussazadeh**	2016	Adult	> 0.05^*^	0.1	0.17	1
**Konar**	2021	Child	NA	NA	NA	NA
**Madsen**	2019	Child	> 0.46	0.06	> 0.09	NA
**Lin**	2017	Child	NA	NA	NA	NA
**Nie**	2022	Mixed	NA	0.48	> 0.37	NA
**Fan**	2021	Mixed	NA	0.35	> 0.93	NA
**Ozgural**	2018	Mixed	0.125	NA	NA	NA

**Notes**: NA: not available; * except in prepontine

**Table 3:** The results of meta-analysis and stratified analysis in outcomes

Results	No. of		Percentage (%)	OR	95%CI	*P* for OR	I [2] (%)	*P* for I [2]
	Studies	EEA/TCA						
**Gross total rection**	7	261/353	87.3/79.1	2.29	[1.15–4.58]	0.019	49.8	0.063
Ault	3	35/26	74.5/60.5	1.84	[0.41–8.16]	0.424	54.9	0.106
Child	1	24/8	85.7/53.3	5.25	[1.21–22.7]	0.027	NA	NA
Mixed	3	202/319	90.2/82.2	2.30	[0.87–6.10]	0.066	63.3	0.066
**CSF leakage**	11	209/59	3.0/1.1	2.80	[2.11–3.72]	< 0.001	24.3	0.212
Ault	5	168/53	3.1/11.0	2.33	[1.71–3.17]	< 0.001	0	0.540
Child	3	21/5	19.2/38.4	4.27	[1.65–11.1]	0.003	0	0.447
Mixed	3	20/1	8.9/0.3	18.18	[4.23–78.2]	< 0.001	0	0.618
**Visual deficit**	7	313/693	4.6/13.6	0.30	[0.26–0.34]	< 0.001	0	0.574
Ault	4	311/668	4.7/14.2	0.30	[0.26–0.35]	< 0.001	14.3	0.321
Child	1	0/2	0/7.1	0.37	[0.02–8.14]	0.525	NA	NA
Mixed	2	2/23	0.9/6.1	0.16	[0.04–0.58]	0.006	0	0.502
**Visual improvement**	6	138/149	78.9/54.4	2.59	[1.67-4.00]	< 0.001	3.6	0.393
Ault	3	33/19	89.2/47.5	3.42	[1.24–9.40]	0.017	0	0.076
Child	1	2/4	28.6/17.4	1.90	[0.27–13.5]	0.522	NA	NA
Mixed	2	103/126	78.6/59.7	2.45	[1.49–4.05]	< 0.001	0	0.792
**Hydrocephalus**	3	131/1179	2/24.3	0.22	[0.02–2.97]	0.256	96.5	< 0.001
Ault	1	119/1162	1.8/25.0	0.06	[0.05–0.07]	< 0.001	NA	NA
Mixed	2	12/17	8.1/8.3	0.68	[0.08–5.52]	0.715	52.4	0.147
**Recurrence**	7	27/88	9.0/18.6	0.44	[0.21–0.92]	0.030	43.9	0.098
Adult	3	5/10	10/17.9	0.41	[0.03–5.24]	0.493	68.6	0.041
Child	2	3/17	7.1/40	0.15	[0.04–0.60]	0.007	0	0.541
Mixed	2	19/61	8.9/16.3	0.54	[0.31–0.94]	0.030	0	0.467
Diabetes insipidus*	8	1444/3098	21.2/60.3	0.47	[0.22–1.04]	0.063	91.1	< 0.001
Ault	5	1336/2864	20.3/60.5	0.45	[0.16–1.26]	0.128	77.7	0.001
Child	1	1/7	7.1/25	0.23	[0.03–2.10]	0.193	NA	NA
Mixed	2	108/234	50.7/62.4	0.61	[0.27–1.38]	0.235	81.8	0.019
**Hypopituitarism**	10	991/1944	14.3/37.2	0.50	[0.28–0.88]	0.016	76.8	< 0.001
Ault	4	796/1572	12.1/33.5	0.40	[0.11–1.39]	0.148	52.8	0.096
Child	3	33/39	23.9/27.3	0.34	[0.12–0.97]	0.043	14.1	0.312
Mixed	3	100/211	44.6/54.4	0.72	[0.49–1.05]	0.089	14.7	0.310
Death*	9	35/244	0.5/4.7	0.64	[0.16–2.54]	0.529	66.5	0.002
Ault	3	30/238	0.4/5.1	0.09	[0.06–0.15]	< 0.001	1.6	0.362
Child	3	2/1	1.4/0.7	1.50	[0.23–9.76]	0.672	0	0.369
Mixed	3	3/5	1.3/1.3	1.02	[0.28–3.70]	0.974	0	0.924
**Stroke**	6	505/596	7.4/11.6	0.58	[0.51–0.66]	< 0.001	0	0.942
Ault	3	497/577	7.5/12.4	0.58	[0.51–0.66]	< 0.001	0	0.971
Child	1	2/5	2/5	0.39	[0.07–2.05]	0.264	NA	NA
Mixed	2	6/14	2.8/3.7	0.70	[0.27–1.84]	0.466	0	0.379
Infection*	4	78/180	1.2/3.7	0.32	[0.24–0.42]	< 0.001	0	0.449
Ault	3	76/172	1.2/3.7	0.31	[0.24–0.41]	< 0.001	11.8	0.322
Mixed	1	2/8	2.3/4.3	0.52	[0.11–2.48]	0.407	NA	NA
**Thrombosis**	3	53/235	0.8/4.9	0.24	[0.09–0.64]	0.004	64.7	0.059
Ault	2	46/218	0.7/4.7	0.25	[0.03–1.85]	0.176	50.8	0.154
Child	1	7/17	7/17	0.37	[0.15–0.93]	0.035	NA	NA

**Notes**: EEA: endoscopic endonasal transsphenoidal approach; TCA: microscopic transcranial approach; NA: not available; *: publication bias was detected

**Table 4:** The significant advantages of a specific surgical approach

	Advantages				Equivalents	
	EEA	TCA				
**Children**	GTR rate	CSF leakage			Visual improvement	
	Recurrence				Visual deficit	
	Hypopituitarism				Diabetes insipidus	
					Death	
					Stroke	
**Adults**	Visual improvement	CSF leakage			GTR rate	
	Visual deficit				Recurrence	
	Hydrocephalus				Diabetes insipidus	
	Death				Hypopituitarism	
	Stroke				Thrombosis	
	Infection					

**Notes**: EEA: endoscopic endonasal transsphenoidal approach; TCA: microscopic transcranial approach

### Quality of reporting assessment

**Fig. 2:**
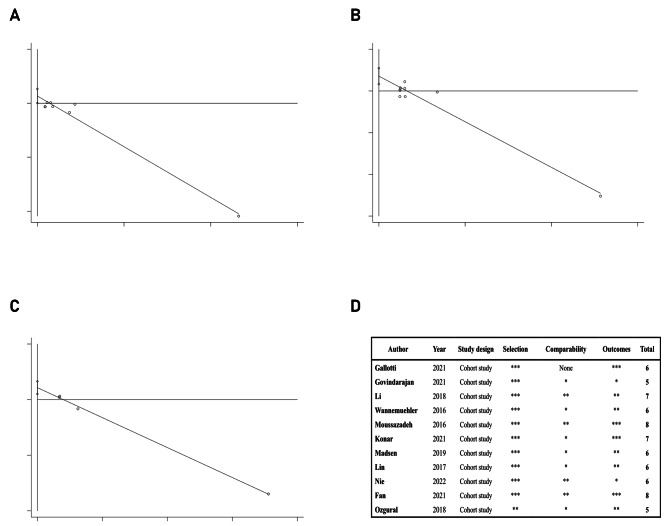
Results of Egger’s test and NOS scores: **A**) diabetes insipidus; **B**) death; **C**) infection; **D**) NOS scores of individual studies

Among 11 enrolled studies, 9 studies were identified high quality, whereas 2 studies were identified low quality. Overall NOS scores for quality assessment were shown in Fig. 2D.

### Surgical outcomes

#### Gross total rection

**Fig. 3:**
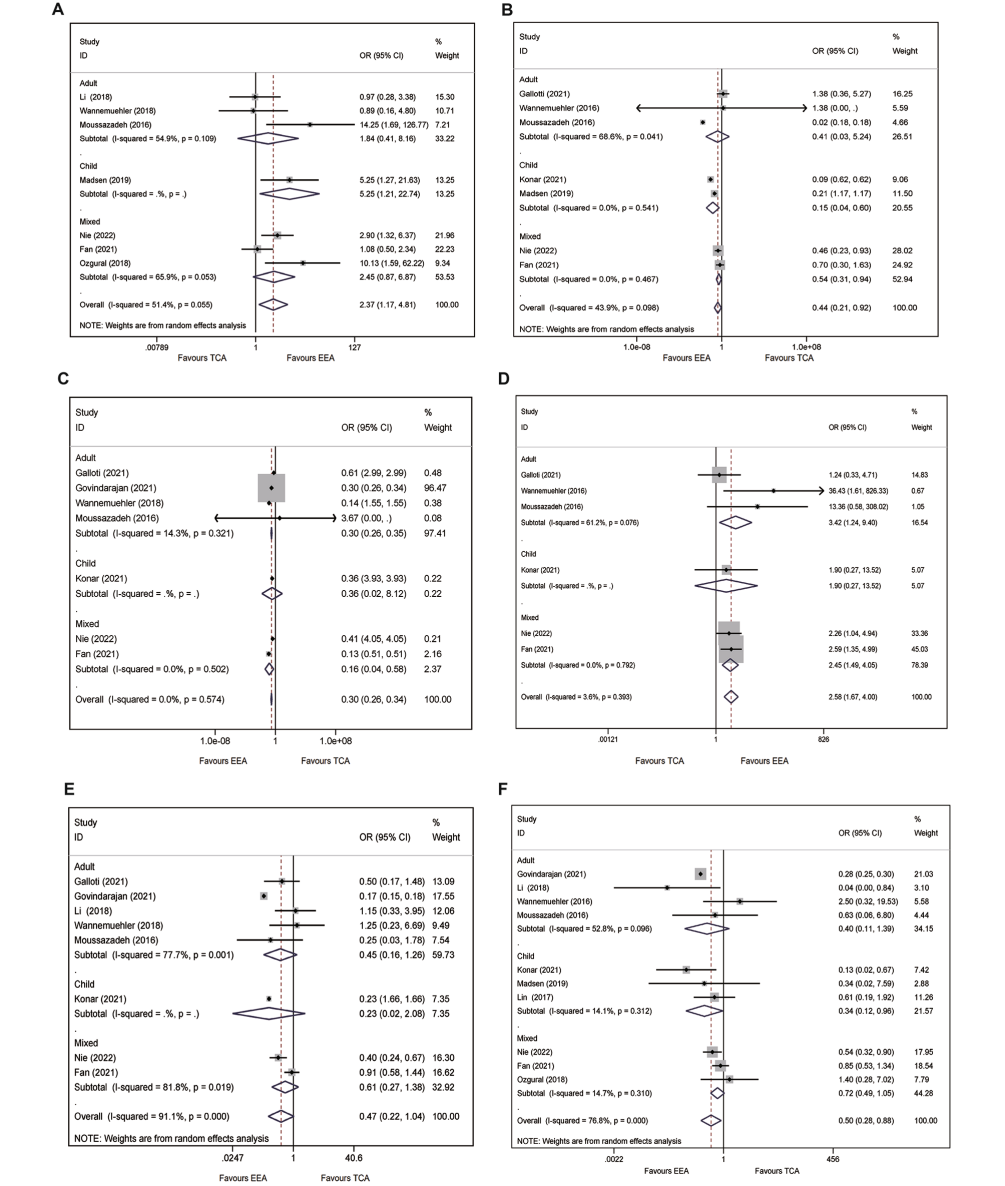
Forest plot for surgical outcomes and endocrine functions: **A**) gross total resection; **B**) recurrence; **C**) visual deficit; **D**) visual improvement; **E**) diabetes insipidus; **F**) hypopituitarism

Compared with TCA, the EEA achieved higher GTR rate in CPs patients (87.3% vs. 79.1%), notably, this superior effect was statistically significant (OR = 2.37, 95%CI: 1.17–4.81; I^2^ = 51.4%, *p* = 0.055, Fig. 3A). Nevertheless, stratified analysis demonstrated that there was no significant difference between the two approaches in adult (OR = 1.84, 95%CI: 0.41–8.16, *p* = 0.424; I^2^ = 54.9%, *p* = 0.106, Fig. 3A) and mixed (OR = 2.30, 95%CI: 0.89–6.10, *p* = 0.019; I^2^ = 63.3%, *p* = 0.066, Fig. 3A) populations, while the EEA showed statistically significant higher GTR rate in child population (OR = 5.25, 95%CI: 1.21–22.74, Fig. 3A).

### Recurrence

The EEA was positively correlated with the reduction of recurrence rate in a pooled analysis (OR = 0.44, 95%CI: 0.21–0.92, *p* = 0.030; I^2^ = 43.9%, *p* = 0.098, Fig. 3B), compared with TCA. The result of stratified analysis showed there were also statistically significant differences in child (OR = 0.15, 95%CI: 0.04–0.60, *p* = 0.007; I^2^ = 0%, Fig. 3B) and mixed (OR = 0.54, 95%CI: 0.31–0.94, *p* = 0.030; I^2^ = 0%, Fig. 3B) populations, nevertheless, this optimistic effect was not observed in adult population (OR = 0.41, 95%CI: 0.03–5.24, *p* = 0493; I^2^ = 68.6%, *p* = 0.041, Fig. 3B).

### Visual deficit

Compared with TCA, EEA significantly reduced the postoperative incidence of new or worsened visual deficit (4.6% vs. 13.6%), and demonstrated a statistically significant difference in this surgical outcome in the pooled analysis with no detected heterogeneity (OR = 0.30, 95%CI: 0.26–0.34, *p* < 0.001; I^2^ = 0%, *P* = 0.547, Fig. 3C). Identically, stratified analysis supported this beneficial effect in adult (OR = 0.30, 95%CI: 0.26–0.35, *p* < 0.001; I^2^ = 14.3%, *p* = 0.321, Fig. 3C) and mixed (OR = 0.16, 95%CI: 0.04–0.58, *p* = 0.006; I^2^ = 0%, Fig. 3C) populations. However, there was no statistically significant difference in child (OR = 0.37, 95%CI: 0.02–8.14, *p* = 0.668, Fig. 3C) population.

### Visual improvement

A percentage of 78.9% and 54.4% patients improved visual outcome after operation with EEA and TCA, respectively. Compared with TCA, the EEA statistically achieved greater likelihood of visual improvement with slight heterogeneity in a pooled analysis (OR = 2.59, 95%CI: 1.67-4.00, *p* < 0.001; I^2^ = 3.6%, *p* = 0.393, Fig. 3D). Notably, in stratified analysis, there were statistically significant difference between EEA and TCA in adult (OR = 3.42, 95%CI: 1.24–9.40, *p* = 0.017; I^2^ = 0%, Fig. 3D) and mixed populations (OR = 2.45, 95%CI:1.49–4.05, *p* < 0.001; I^2^ = 0%, Fig. 3D). No statistically significant difference was observed in child population (OR = 1.90, 95%CI:0.27–13.5, *p* = 0.522, Fig. 3D).

### Endocrine functions

#### Diabetes insipidus

A total of 1,444 and 3,098 patients suffered from postoperative diabetes insipidus (DI), and the incidences of diabetes insipidus were 21.2% and 60.3% in patients underwent EEA and TCA, respectively. However, a pooled analysis indicated there was no statistically significant difference in preventing diabetes insipidus between EEA and TCA (OR = 0.47, 95%CI: 0.22–1.04, *p* = 0.063; I^2^ = 91.1%, *P* < 0.001, Fig. 3E). Similarly, no statistically significantly difference was observed in stratified analysis according to age classification (adult: OR = 0.45, 95%CI: 0.16–1.26, *p* = 0.128; child: OR = 0.23, 95%CI: 0.03–2.10, *p* = 0.193; mixed: 0.61, 95%CI: 0.27–1.38, *p* = 0.235, Fig. 3E).

### Hypopituitarism

A total of 991 (14.3%) and 1,944 (37.2%) patients suffered from postoperative hypopituitarism in EEA and TCA groups, respectively. Compared with TCA, EEA showed statistically significantly lower incidence of hypopituitarism in pooled analysis (OR = 0.50, 95%CI: 0.28–0.88, *p* = 0.016; I^2^ = 76.8%, *P* < 0.001). Likewise, the statistically significant difference was also observed in child (OR = 0.34, 95%CI: 0.12–0.97, *p* = 0.043; I^2^ = 14.1, *p* = 0.312, Fig. 3F). However, subsequent stratified analysis presented opposite results in adult (OR = 0.40, 95%CI: 0.11–1.39, Fig. 3F) and mixed (OR = 0.72, 95%CI: 0.49–1.05, Fig. 3F) populations.

### Complications

#### Cerebrospinal fluid leakage

**Fig. 4:**
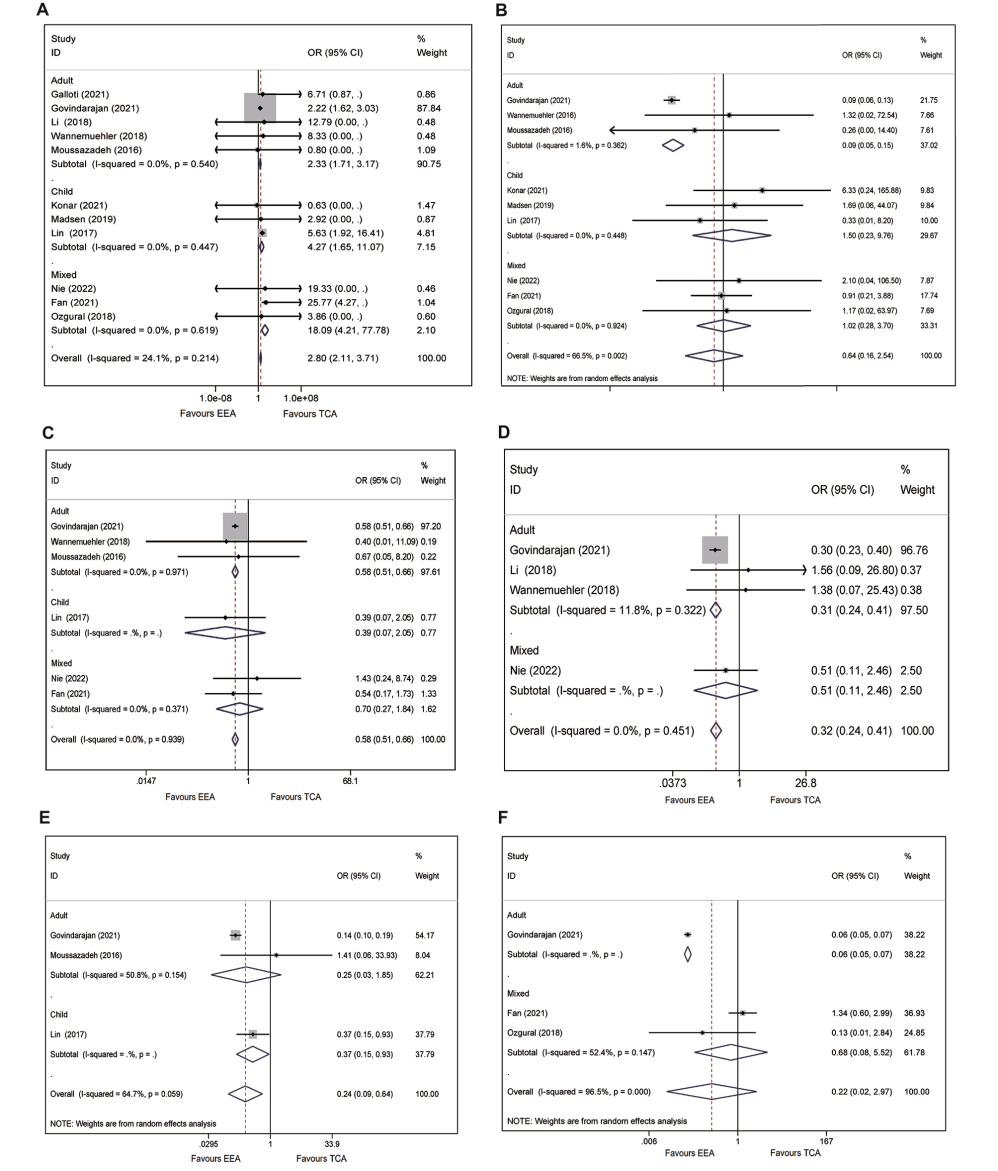
Forest plot for postoperative complications. **A**) CSF leakage; **B**) death; **C**) stroke; **D**) infection; **E**) thrombosis; **F**) hydrocephalus

The incidence of CSF leakage was 3.0% vs. 1.1% in EEA and TCA group, respectively. A pooled analysis indicated significantly higher CSF leakage rate in patients underwent EEA with mild heterogeneity, compared with TCA (OR = 2.80, 95%CI: 2.11–3.72, *p* < 0.001; I^2^ = 24.3%, *p* = 0.212, Fig. 4A). Similarly, stratified analysis showed EEA significantly enhanced the incidence of CSF leakage in all age groups (adult: OR = 2.33, 95%CI: 1.71–3.17; child: OR = 4.27, 95%CI: 1.65–11.1; mixed: OR = 18.18, 95%CI: 4.23–78.2, Fig. 4A).

### Death

The overall all caused death rate were respectively 0.5% and 4.7% in patients underwent EEA and TCA approach. Notably, adult population underwent EEA showed statistically significant difference in reducing death rate, compared with TCA (OR = 0.09, 95%CI: 0.06–0.15, *p* < 0.001; I^2^ = 1.6%, Fig. 4B). Nevertheless, there was no statistically significant difference between EEA and TCA in the pooled analysis (OR = 0.64, 95%CI: 0.16–2.54, *p* = 0.529, I^2^ = 66.5%, *P* = 0.002, Fig. 4B). Stratified analysis showed similar result in child (OR = 1.50, 95%CI: 0.23–9.73, *p* = 0.672, Fig. 4B) and mixed (OR = 1.02, 95%CI:0.28–3.70, *p* = 0.974, Fig. 4B) populations.

### Postoperative stroke

Compared with TCA, the EEA presented a lower incidence (7.4% vs. 11.6%) of postoperative stroke (hemorrhagic or ischemic), and this beneficial effect was also statically significant with no detected heterogeneity in the pooled analysis (OR = 0.58, 95%CI: 0.51–0.66, *p* < 0.001, I^2^ = 0%, *P* = 0.942, Fig. 4C). Subsequent stratified analysis showed identical outcome in adult (OR = 0.58, 95%CI: 0.51–0.66, *p* < 0.001; I^2^ = 0%, *p* = 0.971, Fig. 4C). However, there were no statistically significant differences between EEA and TCA in child (OR = 0.39, 95%CI: 0.07–2.05, *p* = 0.246, Fig. 3C) and mixed (OR = 0.70, 95%CI: 0.27–1.84, *p* = 0.466, Fig. 4C) population.

#### Infection

A total of 78 and 180 patients (1.2% vs. 3.7%) suffered from postoperative infection in EEA and TCA group, respectively. Compared with TCA, EEA significantly reduced the incidence of postoperative infection with detected heterogeneity in pooled analysis (OR = 0.32, 95%CI: 0.24–0.42, *P* < 0.001; I^2^ = 0, Fig. 4D). On the contrary, stratified analysis did not support this beneficial effect in mixed population (OR = 0.52, 95%CI: 0.11–2.48, *p* = 0.407, Fig. 4D).

### Thrombosis

The incidences of thrombosis (deep venous thrombosis or pulmonary embolism) in EEA and TCA group were respectively 0.8% and 4.9%. Compared with TCA, EEA showed statistically significant difference in preventing thrombosis a pooled analysis (OR = 0.24, 95%CI: 0.09–0.64, *p* = 0.004; I^2^ = 64.7%, *P* = 0.059, Fig. 3E). Stratified analysis showed statistically significant difference in child population (OR = 0.37, 95%CI: 0.15–0.93, *P* = 0.035, Fig. 3E), while the difference in child population was not statistically significant (OR = 0.25, 95%CI: 0.03–1.85, *P* = 0.175; I^2^ = 50.8%, *p* = 0.154, Fig. 4E).

#### Hydrocephalus

The lower incidence of hydrocephalus (2.0% vs. 24.3%) was observed in adults underwent EEA approach, compared with TCA. Nevertheless, this noticeable difference was not statistically significant with substantial heterogeneity in a pooled analysis (OR = 0.22, 95%CI: 0.02–2.97; I^2^ = 96.5%, *P* < 0.001, Fig. 4F). Stratified analysis showed significantly difference in adult (OR = 0.06, 95%CI: 0.05–0.07, *p* < 0.001, Fig. 4F) population, whereas the difference was not statistically significant in mixed population (OR = 0.68, 95%CI: 0.08–5.52, Fig. 4F).

#### Publication bias

The results of Egger’s test in the assessment of publication bias demonstrated three outcomes in the pooled analysis may carry a possibility of threat to the validity of meta-analysis, suggesting that the publication bias may affect the evidence-based decision making in recommendations of EEA and TCA in preventing postoperative diabetes insipidus, death and infection (Fig. 2A, B, C).

## Discussion

Being one of the most challenging problems for neurosurgeons, CPs present an urgent requirement for neurosurgeons to provide high quality evidence for clinical practice and evidence-based decision-making for its management [2, 35]. Previous meta-analyses have reported only a pooled single-proportion and mostly included single series studies in their literature searches rather than comparative research, suggesting that the included studies in these meta-analyses may be clinically heterogeneous [13, 36]. Moreover, pooling single series research with different methodologies may present a potential source of bias for mismatching patients with different clinical characteristics, proficiency of operators, and originating from different countries, especially when comparing the pooled effect size between the EEA and TCA groups. Therefore, we performed a systematic review and meta-analysis by pooling the results of the comparative studies to provide more comprehensive and valid evidences on the clinical practice of CPs. This meta-analysis, which was based on 12,212 participants, is the largest comparative meta-analysis that focused on the safety and effect of the EEA and TCA in the management of CPs. To minimize the clinical heterogeneity, we only included observational studies that directly compared the effects and safety of the EEA and TCA for treating CPs. Notably, in consideration of the different age populations being the main source of clinical heterogeneity, which may affect the reliability of the evidence, we performed a stratified analysis to limit the potential influence of bias caused by ages.

Previous studies have suggested that the GTR is associated with a significantly lower recurrence rate compared with STR, resulting in the achievement of GTR being generally accepted as the primary objective for surgical management in CP patients [4, 5, 10, 37]. Controversially, studies investigating whether GTR differs from STR + RT (subtotal resection *plus* adjuvant radiation therapy) have reported comparable long-term survival rates and postoperative complications [37, 38]. However, despite the limited choice of patients to select a specific medical center that offers the radiation therapy service, feasibility of the selected patients whose tumor could easily achieve GTR, and undetermined potential iatrogenic injury by radiation therapy, especially in children, GTR should still be recommended as the main goal for neurosurgeons when resecting CP tumors [2, 13, 37, 39]. In this study, the aggregated data from existing publications showed a significant association between the EEA and a higher GTR rate in a pooled analysis compared with TCA (OR = 2.37, 95%CI 1.17–4.81, *p* = 0.017). This superior effect is consistent with previous meta-analyses and supports the fact that EEA can provide a clear and broad field of vision, confirm the relationship between the pituitary stalk and tumor, protect the superior pituitary artery, and prevent the destruction of brain tissue via microsurgical methods.

Although the pooled effect supports the recommendation of EEA in patients with CP for attaining a higher GTR rate and is consistent with a previous meta-analysis published in 2011, the results of the stratified analysis could provide more individualized evidence for clinical practice [13]. In the stratified analysis, the EEA did not show any significant difference in the adult (OR = 1.84, 95% CI 0.41–8.16, *p* = 0.424) and mixed (OR = 2.45, 95% CI 0.87–6.87, *p* = 0.066) groups for achieving a higher GTR rate as compared with TCA. Generally, although a higher GTR rate was commonly considered as an advantage of the EEA by most published researchers, the results from our study and those of several recent studies did not support this superiority, suggesting that the possibility of the surgical technique of the TCA in neurosurgeons might have improved along with the development of other surgical techniques [11, 40]. Moreover, Younus et al. confirmed that the higher GTR rate was significantly achieved by senior specialists than surgeons with limited clinical practice (71% vs. 47%, *p* < 0.05), suggesting that the GTR rate in EEA might increase as neurosurgeons improve their surgical process using the EEA in the next decade [41]. Contrary to the adult group, the child group exhibited a significantly higher GTR rate in the EEA group (OR = 5.25, 95% CI 1.21–22.7, *p* = 0.027). However, only one study was included in calculating the effect size. A current research identified the safety of the EEA in pediatric patients, eliminating the concerns of several pioneers regarding the potential influence of the EEA affecting the midfacial development of children. Our result is similar to that of a previous meta-analysis, which reported a notably high-pooled GTR proportion (75.8%) in pediatric patients under the EEA, suggesting that the recommendation of the EEA in children should be positive. Moreover, although a few studies identified significantly different incidences of complications between the earlier and latter groups, published series in this field did not sufficiently report the difference between the earlier and latter cohorts [41, 42]. Therefore, although the EEA has been considered the first-line treatment option, evidence of the association between the EEA and GTR rate among adults is limited, and further research is needed to identify long-term outcomes. In conclusion, given these considerations, the formulation of the surgical strategy should follow the guidance of the preoperative assessment, which must include the clinical characteristics, such as tumor texture, magnetic resonance imaging (MRI) findings, presenting symptoms, and endocrine function.

Pioneers have confirmed that the higher achievement of GTR is commonly accompanied by a significantly lower recurrence rate in primary CP resection [9, 43]. In our study, although EEA showed a significant reduction in recurrence, as previously mentioned, the EEA achieved a higher GTR rate in the pooled analysis. These results strongly support the recommendation of the EEA based on its superiority in GTR and avoiding recurrence in primary CP resection. However, unlike the findings of a previous meta-analysis, the stratified analysis in this study significantly supported the superiority of the EEA in the child population (OR = 0.15, *p* = 0.007) [8, 12]. Notably, contrary to our study (OR = 1.84, *p* = 0.424), a previous meta-analysis reported a significant superiority of the EEA in GTR in the adult population [44]. These controversial results might be attributed to the fact that the previous meta-analysis only enrolled one single series rather than comparative series and the STR + RT was recently accepted as an equivalent treatment with a similar recurrence rate as compared with achieving GTR [38, 45].

With the revolutionized application of the nasoseptal flap in skull base reconstruction, the CSF leakage rate is significantly reduced in endoscopic skull base surgery as compared with the traditional flap [46]. In our study, the EEA showed a significant association with CSF leakage in a pooled analysis (OR = 2.80, 95% CI 2.11–3.72, *p* < 0.001) and stratified analysis (adult, OR = 2.33, 95%CI 1.71–3.17; child, OR = 4.27, 95% CI 1.65–11.1; mixed, OR = 18.18, 95% CI 4.23–78.2). Optimistically, the pooled 3.0% incidence of the postoperative CSF leakage is generally acceptable, considering the EEA showed a significant reduction in the postoperative infection rate (overall, 0.32, *p* < 0.001; adult 0.31, *p* < 0.001). On the other hand, a recent prospective, randomized controlled trial determined that the placement of perioperative lumbar drainage could significantly decrease the incidence of postoperative CSF leakage as compared with the control group (OR = 3.0, 95% CI 1.2–7.6, *p* = 0.017) [47]. Furthermore, this study might change the clinical practice guidelines of the perioperative management of endoscopic skull base surgery, providing more data to future research.

The visual outcome is commonly accepted as the focus of neurosurgeons when it comes to assessing the operation results and quality of life. In this study, the pooled analysis identified the significant advantage of the EEA not only in improving visual outcomes but also in preventing visual deficits, especially in adults (only one study involving a child population was enrolled). These results were consistent with those of previous meta-analyses. Moreover, a meta-analysis on a mixed population conducted by Ricardo et al. showed similar results, suggesting the potential advantage of the EEA in children [13, 44, 48]. Recently, Qiao et al. have determined two independent risk factors (i.e., tight adhesion and larger tumor volume) for postoperative visual field defects and confirmed that the use of intraoperative visual evoked potential (VEP) reliably guided neurosurgeons to minimize intraoperative injury of the optic chiasma and prevent postoperative visual deterioration in the adult population [3]. Therefore, despite the uncertainty of the surgical effect of the EEA in children, the EEA procedure *plus* intraoperative VEP monitoring could be strongly recommended as the preferred option when formulating an individualized surgical strategy for adult patients with presenting symptoms of visual deficits or compressed optic nerve.

Generally, the EEA presents a more direct visualization of the pituitary stalk and minimizes the need for cerebral retraction and the manipulation of neurovascular structures, providing a theoretical and clinical reduction in postoperative endocrine results (diabetes insipidus and hypopituitarism). Nevertheless, in this study, unlike previous meta-analyses, no significant differences in diabetes insipidus and hypopituitarism were observed between the EEA and TCA in both the child and adult populations, except that the EEA significantly decreased postoperative hypopituitarism in the child population (OR = 0.34, *p* = 0.043)^8,11,48^. These controversial results might be attributed to the detected publication bias (*p* = 0.046), various classifications of endocrine dysfunction (hypothyroidism, hypogonadism, adrenal insufficiency, and growth hormone deficiency), different growth directions of the CPs, and the definition of DI duration (temporary or permanent). Fan et al. assessed the different surgical and endocrine outcomes of the EEA and TCA in CP resection in adults and identified the contrary results of postoperative hypopituitarism between the T-CP (TCA preceded EEA, *p* = 0.016) and Q-CP (EEA preceded TCA, *p* = 0.008) patients, respectively [30]. Given these considerations, it can be suggested that future original studies, especially prospective studies, should formulate a comprehensive statistical scheme, which could increase reliability and precision for clinical guidelines and evidence-based decision-making.

The stratified analysis of the complications in our study showed that, for adults, the EEA reduced the postoperative infection, stroke, hydrocephalus, and all-cause death rate. However, for pediatric patients, the EEA only decreased postoperative thrombosis. These superiorities of the EEA might be attributed to its advance in the surgical field, minor injuries to the brain tissue, and shorter LOS. Notably, the higher CSF leakage rate in the EEA group in all populations, as previously mentioned, did not sufficiently cause a higher infection and death rate, suggesting that this complication should not be a main concern when surgeons were about to determine the individualized surgical approach.

Additionally, original studies in this research field occasionally reported incomparable outcomes. For example, several studies have proven the median of LOS, while others preferred the mean number or interquartile range. However, the absence of accurate definitions of clinical outcomes, such as hypopituitarism and DI in original studies, may lead to less precise results. Therefore, the lack of report of detailed outcomes limited our study to provide sufficient evidence and further analysis. More importantly, the absence of radiologically characteristic of CP may lead a potential incomparability between two different interventions. Therefore, we recommend that further study should accurately report the relevant data to improve the quality of evidence in this field. Furthermore, we were unable to sufficiently compare the association of the surgical approach and tumor volumes, various QST classifications, and specific tumor texture. Thus, future research is needed to investigate these clinical heterogeneities.

## Conclusions

The advance and prevalence of EEA significantly improved several postoperative surgical outcomes, endocrine functions, and complications in primary CP resection as compared with the traditional TCA. For adults, the EEA showed significant superiority in decreasing postoperative hydrocephalus, stroke, infection, mortality, and visual deficit. Moreover, for pediatric patients, the EEA was associated with a significantly higher GTR rate, less recurrences, and lower hypopituitarism rate. The only significant disadvantage of the EEA was the higher CSF leakage rate. Nevertheless, despite the higher CSF leakage rate, the EEA unexpectedly showed a significantly lower rate of postoperative infection, suggesting that this inferior effect could not be considered as a main concern when surgeons were determining the EEA as the optimal approach for primary CP resection.

### Electronic supplementary material

Below is the link to the electronic supplementary material.

Supplementary Material 1

## Data Availability

No datasets were generated or analysed during the current study.

## Declarations

### Provenance and peer review

Not commissioned, externally peer-reviewed.
