# Supplementary material for: Endoscopic endonasal transsphenoidal approach improves endocrine function and surgical outcome in primary craniopharyngioma resection: a systematic review and meta-analysis
Source: World J Surg Oncol. 2024 May 24;22:137. doi: 10.1186/s12957-024-03411-8 (PMC11118612; doi:10.1186/s12957-024-03411-8)
Supplement: Supplementary file 1 — Supplementary Material 1 [file 12957_2024_3411_MOESM1_ESM.docx]

**Title page**

**Endoscopic endonasal transsphenoidal approach improves endocrine function and surgical outcome in primary craniopharyngioma resection: A Systematic Review and Meta-Analysis**

**Brief title: EEA in primary craniopharyngioma resection.**

**Authors: Shuang Li, ^1, #^ Youfan Ye, ^2, #^ Chuansheng Nie, ^1^ Xing Huang, ^1^ Kaixuan Yan, ^1^ Fangcheng Zhang, ^1,^** * **Xiaobing Jiang ^1,^** * **and Haijun Wang ^1,^** *

**^1^** Department of Neurosurgery, Union Hospital, Tongji Medical College, Huazhong University of Science and Technology, 1277# Jiefang Avenue, Wuhan, Hubei, 430022, China.

**^2^** Department of Ophthalmology, Union Hospital, Tongji Medical College, Huazhong University of Science and Technology, 1277# Jiefang Avenue, Wuhan, Hubei, 430022, China.

**^#^** There authors contributed equally to this work.

* Correspondence: Fangcheng Zhang, [zfcwhuh@gmail.com](mailto:zfcwhuh@gmail.com); Xiaobing Jiang, [jxb917@126.com](mailto:jxb917@126.com); Haijun Wang, [wwanghhaijjun@sina.com](mailto:wwanghhaijjun@sina.com).
